# Trialogue Meetings: Engaging Citizens and Fostering Communities of Wellbeing Through Collective Dialogue

**DOI:** 10.3389/fpsyg.2021.744681

**Published:** 2021-12-20

**Authors:** Liam Mac Gabhann, Simon Dunne

**Affiliations:** ^1^School of Nursing, Psychotherapy and Community Health, Dublin City University, Dublin, Ireland; ^2^School of Psychology, Dublin City University, Dublin, Ireland

**Keywords:** open dialogue, trialogues, wellbeing, participation, citizenship, Trialogue Meetings

## Abstract

Community-based participatory approaches are widely recognized as valuable methods for improving mental health and well-being by enabling a greater sense of liberty among participants, through the development of equitable policies and practices, which accommodate a range of diverse perspectives. One such approach, “Trialogue Meetings,” has been found to encourage disclosure and dialogue surrounding mental health, facilitate the growth and development of communities in relation to people’s experience of mental health difficulties, service provider and community response. Emerging in the 1990s because of perceived and felt inequitable relations between people with lived experience of mental health difficulties, family members of people with mental health difficulties and professionals providing mental health service provision. This approach has been shown to successfully reduce stigma and discrimination and improve relations between stakeholders in community and mental health care settings. Trialogue Meetings incorporate Open Dialogue methods to allow multiple stakeholder groups to participate in conversations around a given topic and enable the creation of a common language and mutual understanding. Trialogue Meetings have added benefits of allowing individuals to express themselves better, gain a sense of relationality and community with others and address predetermined power hierarchies with prescribed responses to people’s experiences. In this perspective, we present an outline for Trialogue Meetings as a medium for enhancing wellbeing, providing a transformative empowering process for deliberate discursive practice and engaging citizens through sustained collective dialogue.

## Introduction

Community-based participatory approaches are widely recognized as valuable methods for improving mental health and well-being (Prilleltensky and Nelson, 2002; Kidd et al., 2014). They are typically designed to strengthen networks within organizations and communities (Heath, 2007) and have the potential for systemically excluded groups to address power imbalances and give voice to diverse perspectives (Kidd et al., 2014). Furthermore, they are specifically designed to create participatory collaborative processes, through which more integrated approaches to sustaining mental health and well-being, and communities themselves, can develop (Reason and Bradbury, 2008).

Trialogue Meetings provide such a participatory community approach. They draw upon Open Dialogue approaches (Bakhtin, 1981) that enable the creation of a common language and mutual understanding around given topics (For further details regarding this approach, see MacGabhann et al., 2012). Trialogue Meetings have persevered in mental health communities. In this context, they have been recognized for their potential to enable transformative dialogue in relation to contentious issues amongst people with mental health problems, family members/supporters of people with mental health problems and mental health professionals. Due to these successes, Trialogue approaches have begun to be applied more widely to participatory research and community/organizational development. In this paper, we discuss the background to Trialogue Meetings in mental health and their emerging application to diverse areas. We also describe our vision for Trialogue Meetings as a tool for engaging citizens in democratic and equitable discussions that foster wellbeing and conditions for mutual understanding surrounding particular topics of enquiry.

## Background to Trialogue Meetings in Mental Health Communities

Trialogue Meetings emerged in Germanic speaking countries in the late eighties. They were instigated by Dorothea Buck, a survivor of concentration camps and dubious psychiatric practices in Nazi Germany. She sought to create neutral community-embedded spaces for discourse surrounding mental health, where mental health service users, family members and mental health professionals could engage with each other on an equal footing, where hierarchical power structures or dominant knowledge expertise carry no added value (Lehmann, 2015). Following these ground-breaking “Psychosis Seminars” in Hamburg, the first “Vienna Trialogue” was established in 1994 and, since then, the approach has been embraced by over 150 groups across Germany, Austria and Switzerland (Amering et al., 2002, 2012). Trialogue Meetings and related approaches have subsequently emerged in several other countries over the last two decades, including the United Kingdom, China, Poland, Turkey, Trinidad, Toronto United States and Ireland (MacGabhann et al., 2012, 2018).

**FIGURE 1:**
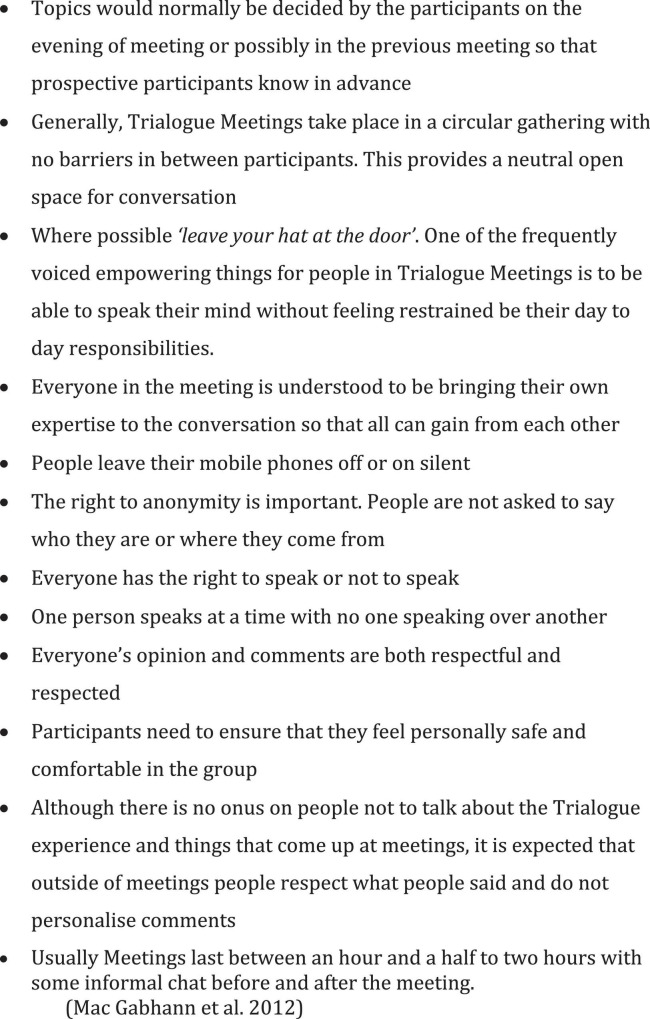
Trialogue meeting ground rules.

Early Trialogue Meetings typically focused on the topic of psychosis (Bock et al., 2000). Over time, their focus expanded to cover broader topics surrounding mental health. The meetings themselves take place in neutral venues, outside of family or mental health provider settings. Trialogue draws on Open Dialogue approaches where participants have to be willing to engage in the dialogue and enter into a joint action that brings them together in a temporary mutual world experience (Bakhtin, 1981). Equally, in this dialogue they can choose to participate in silence. Dialogue topics are either agreed in advance, or at the beginning of meetings, and one participant facilitates the dialogue within some simple dialogical ground rules for the group (see Figure 1).

Underpinned by a social constructionist philosophy (e.g., Gergen, 1985), Trialogue Meetings embrace the collective construction of a shared reality that is mutually acceptable and accessible to all participants. There is no exclusivity of expert knowledge or power in Trialogue, and the diverse experiences and perspectives of all participants on the topic of enquiry carry equal weight (MacGabhann et al., 2012). The combined expertise of these diverse voices provides a unique wealth of collective knowledge to which these individuals would not otherwise be exposed. Furthermore, they provide a platform for “vital” and “transformative” self-expression, where they facilitate participants to communicate with others freely and truthfully (Dunne et al., 2018a).

## Research and Development With Trialogue Meetings Among Mental Health Communities

Although there have been consistent anecdotal reports indicating that engaging in Trialogue Meetings can be positive and transformative experiences (Bock et al., 2000; Amering et al., 2002, 2012; Bock and Priebe, 2005), there has been limited formalized research on the impact that Trialogue Meetings have had on participants. One small-scale mixed methods study by von Peter et al. (2015) showed that Trialogue Meetings facilitated knowledge production and understanding and were characterized by aspirations of good will and openness. Additionally, Ruppelt et al. (2015) found that Trialogue participants had more positive attitudes toward mental health symptoms and experienced less anxiety and greater empowerment.

Since these earlier research experiences, we have used Trialogue Meetings as part of a participatory action research methodology in a mental health setting (Dunne et al., 2018a,b; MacGabhann et al., 2018), where Trialogue Meeting participants guided decision-making in all aspects of research design. Through this research program, we have demonstrated that Trialogue Meetings can increase knowledge/awareness and encourage disclosure/dialogue surrounding mental health. We have also found that they can successfully reduce stigma and discrimination and improve relations between community mental health stakeholders. Furthermore, we have found that Trialogue Meetings can be a sustainable community-building approach to citizen engagement, wellness, diversity and empowerment. Where established participatory relations are present, they have continued to grow and develop in many locations after a project development team has handed over responsibility to local communities themselves.

Following the above, many proponents of Trialogue Meetings have sought to develop and adapt the approach further. Once immersed in the dialogical way of being facilitated by this approach, these “Trialoguers” have adapted it for the purposes of education, service improvement and alternative approaches to research. The approach has been utilized in organizational and community development (MacGabhann et al., 2010; Kenny et al., 2020) to engage relevant stakeholders with diverse perspectives in transformative dialogue and enable shifts in attitudes and practices for the purpose of mental health service improvement and recovery education. For instance, the Mental Health Trialogue Network Ireland used Trialogue to bring service providers, service users and family members together to collectively lead improvements in their local mental health service (MacGabhann et al., 2010). Where strong power dynamics between groups prevails and difficult to reach communities are being accessed, Open Dialogue through Trialogue is gaining momentum as a research methodology (e.g., Piippo and MacGabhann, 2016; Proudfoot et al., 2019). Whilst Trialogue Meetings have served mental health communities for several decades, there is still limited published research as to their impact and none that counters their espoused benefits. Nonetheless, further research needs to be undertaken to both critique and fully understand the potential benefits of this approach.

## Trialogue Meetings as a Process for Engaging Citizens in Deliberate Dialogue for Enhancing Wellbeing

That Trialogue has persisted in mental health settings over three decades offers some testament to its usefulness as an approach. Trialogue Meetings have provided a deliberate democratic process for citizen engagement and empowering dialogue in this context that has fostered personal and community transformation in terms of thinking, attitudes, behavior and collective meaning in relation to mental health topics and experiences of wellbeing. As the research above demonstrates, mental health communities themselves have thus far validated this approach within this context, as a useful process of engagement, community development, service improvement and means to specifically tackle stigma and discrimination toward disempowered groups. With this “proof of concept” for Trialogue Meetings in mental health settings, we envision that the approach may be equally applicable to other societal contexts where marginalization, gender inequality, racism, exclusion, questionable citizen status, diverse perspectives, and significant power dynamics prevail. Interestingly, historical critiques posit that disability as an overarching label has been used to justify inequality for women and minority groups (Baynton, 2001). So the challenge within mental health may well apply to all of the above groups.

Many of the challenges inherent among mental health communities are not uniquely the preserve of these groups. Pre-existing power dynamics, perceived dominance of certain expertise and exclusion of minority citizen perspectives and experiences prevail across society where diversity exists and, in particular, where hegemonic practices are embedded in policy, academia and organizational culture and service provision. Indeed, the unidirectional application of expert knowledge from academics and service providers toward “non-expert citizens” has been shown to be problematic, as the benefits of such knowledge may not be as helpful for their wellbeing as it is for the purveyors of that knowledge (Prior, 1991; Faulkner and Thomas, 2002). For instance, Torfing et al. (2019) have identified that the two predominant paradigms of public service organizational culture and provision (as a legal authority or as a service provider) perpetuate disempowerment and passivity among citizens rather than facilitate citizens to define and solve the shared problems and common tasks in society.

In attempts to address such disempowerment and passivity, there have been consistent calls for more collaborative approaches to knowledge development and citizen engagement in research and development. In particular, there has been an evolving discourse surrounding Public and Patient Involvement (PPI) in health and social care and “co-design”/“co-creation” in public services, research and development internationally (e.g., Staniszewska et al., 2017; Baptista et al., 2019; Torfing et al., 2019). Arguably, this suggests a growing shift toward greater citizen involvement. Nonetheless, according to Grotz and Poland (2020), there remains disconnection between aspirations of collaborative engagement policy and what actually constitutes engaging different perspectives in the decision-making of research and development that incorporates such approaches, with no clarity on the extent to which such involvement should lead to discernible practical changes. Furthermore, there have been documented attempts to implement a “co-creation” paradigmatic approach in research and development (e.g., relating to public services; Chadwick, 2011; Echeverri and Skålén, 2011) that have failed.

In contrast, we believe the Trialogical approach to Open Dialogue provides a potential participatory collaborative process through which connections can be made between individuals with diverse perspectives, where the co-creation of collective meaning is possible and mutuality and diversity is harnessed for community transformation. As an approach, there is no reason why it cannot be applied to diverse areas across the spectrum of society where citizen engagement toward enhanced wellbeing and social justice are desired outcomes. The following discussion explores the nuances of how Trialogue seems to work as a participatory approach to citizen engagement that can enhance wellbeing and social justice.

## Discussion

What is it about Trialogue that suggests it may be harnessed as an effective participatory approach to citizen engagement? We believe that there are key features of Trialogue Meetings that point toward its inherent democratic nature. Firstly, Trialogue Meetings can facilitate a leveling of pre-existing power structures through an open dialogical forum where hierarchies no longer exist. More specifically, as with other forms of Open Dialogue, Trialogue Meetings offer an anonymous space where individuals from diverse contexts are enabled to abandon their normal roles and participate in a democratic form together with individuals who typically hold a different level of power (MacGabhann et al., 2018). The social constructionist philosophy (e.g., Gergen, 1985) underpinning this approach also specifically recognizes that people construct their reality individually and that there are multiple, yet equally valid, socially constructed perspectives on such “realities.” Indeed, ground rules for Trialogue Meetings dictate the mutual appreciation that everyone in the conversation brings their own expertise to a given situation (see MacGabhann et al., 2012), thereby redistributing power in a favorable fashion in contexts where power differentials may exist. In this way, Trialogue Meetings are tailor-made for citizen engagement in contexts such as public health and social policy as a means to include the general public and individuals from minority contexts, who often feel excluded from considerations of citizenship (Vervliet et al., 2019; Clayton et al., 2020). Another feature of Trialogue Meetings which supports its potential as an approach to citizen engagement is its focus on establishing collective dialogue through suspension of our normal preconceived assumptions and exploration of how these preconceived assumptions may differ. Such processes of suspending one’s assumptions and exploring perspectival divergences can be challenging, and lead to an initial guarded series of exchanges. Nonetheless, we have found that this may dissipate over time and give way to open free-flowing conversations in which Trialogue participants explore different roles and ideas and express confidence in not knowing where the conversation might lead (Dunne et al., 2018a).

What is it about Trialogue that creates such a transformative experience? We believe that the inter-subjectivity between Trialogue participants creates a relational co-constructed way of knowing (Habermas, 2003; Kemmis, 2008) between them through dialogue. Habermas’s (1976) idea of “social validity,” where active dialogue results in a collective consensus of truth and understanding, resonates with this sense of collaborative agreement achieved in Trialogue. Social validity as an outcome of the dialogical process has to meet four criteria; comprehensiveness, truth, rightness and authenticity. Perhaps this relational knowing, generated through collective consensus-building dialogue, is party to mutual transformation occurring in Trialogue. Habermas (1987) posits a theory of communicative action that supports the primacy of communication in maintaining a balance of power in relation to the exploitation of knowledge, toward the advancement of an individual or specific group in society. In essence, communicative action if dialogue prevails can accommodate the preservation of peoples’ “life worlds,” even within contexts with oppressive or hierarchical power structures in relation to knowledge production and communication practices. For Habermas, the creation of a consensual dialogue toward mutual truth is the aim of communicative action; an aim which seems to be possible to achieve through Trialogue Meetings. Furthermore, the emergence of a collective dialogue has the potential to create a spontaneous activity where mutual truth can be created between individuals as a group outcome (Shotter, 1997). These dialogical moments seem to constitute a dialogical space and understanding, which no longer requires words, moving from explicit knowledge to implicit knowledge as an embodied experience (Stern, 2004). Bohm (2004) argues that, if we learn to suspend our initial assumptions in favor of group creativity in this way, we may be able to engage in a dialogue (rather than a “conversation”) between individuals with diverse perspectives that can accommodate diverse worldviews. He contends that, to generate anything new, a collective dialogue has to ensue where we move beyond our predetermined thoughts, exploring our incoherence, and discrepancies in our points of view. Trialogue Meetings facilitate this approach, thereby having the potential to appropriately represent and validate diverse perspectives and voices in an equitable manner in forums where social justice considerations are a priority. In this context, Trialogue Meetings may also inherently foster empathic communication through an opportunity to understand and appreciate the perspectives of individuals involved in the conversation (MacGabhann et al., 2018).

Trialogue Meetings can also facilitate individuals to express themselves freely and truthfully; here, the opening up the possibility of a collective dialogue appears to enable a freer flowing conversation through which participants can safely explore different roles and ideas, open to an unknowing process of where the conversation may lead (Dunne et al., 2018a). Here, there is a correspondence between the “transformative” power of self-expression in Trialogue Meetings and Maurice Merleau-Ponty’s (1962) ideas that speech animates or vitalizes ideas, enables an individual’s thoughts to be brought to completion and brings ideas to life through the bodily expression of gesture. This can lead to the creation of a new “vital” and “shared” language in Trialogue Meetings, facilitating a shared understanding or meaning between participants and enabling a sense of universality or collectivity to emerge through dialogue. We have found that this process of dialogic self-expression may lead to positive outcomes for Trialogue participants in relation to their well-being such as lower anxiety (Dunne et al., 2018a).

Although these inherent features of Trialogue demonstrate its potential for social justice, citizen engagement and well-being enhancement, Trialogue is not a natural way of being and has to be learned. Bohm (2004) describes how the initial “incoherence” of dialogue only surrenders to coherent expressions of a collective and shared sense of meaning through practice. We have found that the development of dialogic skills and adoption of the rules of engagement for Trialogue Meetings may be critical elements that enable a more coherent shared perspective and collective dialogue to take place (Dunne et al., 2018a). For instance, sensitivity regarding “when to come in” may be aided by the rules of engagement for Trialogue such as the option for anonymity and the opportunity to speak without interruption.

## Conclusion

The time of unidirectional “evidence based practice” and “hierarchical expertise” is increasingly losing its hegemonic position, at least in relation to health and social care research and practice. In contrast, PPI and co-creation, where the value of diversity of experiences and “end-user” knowledge is increasingly being recognized. Wider social policy also provide testimony to a paradigm shift in how citizens can and must be involved in determining how they contribute to the discourse on their own wellbeing; for example, in relation to determinants of health and reimagining responses to ill health (Puras, 2020).

To rise to complex challenges associated with systemic change and accommodation of pluralistic truths, we need a way of engaging with difference. We need a participatory approach that can accommodate predetermined assumptions, embedded beliefs, traditional ways of knowing and citizen diversity. We need a new process that will capitalize on the collective expertise, yet overcome historical truths and ways of being toward new paradigms and social evolution. The Trialogue approach offers such an approach; one that is reflexive and has been shown to offer a resilient form of citizen engagement. We contend that Trialogue has important applications beyond its community of origin, as an approach to social justice and engagement, which can enhance, and explore conceptualizations of what constitutes, citizen wellbeing.

## Data Availability Statement

The original contributions presented in the study are included in the article/supplementary material, further inquiries can be directed to the corresponding author/s.

## Author Contributions

LM was led author writing initial multiple drafts of each section of the manuscript in discussion with SD. SD contributed to the discussion design and contributed to each section of manuscript with structural and conceptual edits. Both authors contributed to the article and approved the submitted version.

## Conflict of Interest

The authors declare that the research was conducted in the absence of any commercial or financial relationships that could be construed as a potential conflict of interest.

## Publisher’s Note

All claims expressed in this article are solely those of the authors and do not necessarily represent those of their affiliated organizations, or those of the publisher, the editors and the reviewers. Any product that may be evaluated in this article, or claim that may be made by its manufacturer, is not guaranteed or endorsed by the publisher.
